# Long Noncoding RNA HOTAIR Functions as a Competitive Endogenous RNA to Regulate Connexin43 Remodeling in Atrial Fibrillation by Sponging MicroRNA-613

**DOI:** 10.1155/2020/5925342

**Published:** 2020-11-17

**Authors:** Weiran Dai, Xiaoying Chao, Shanshan Li, Shuang Zhou, Guoqiang Zhong, Zhiyuan Jiang

**Affiliations:** ^1^Department of Cardiology, The First Affiliated Hospital of Guangxi Medical University, Guangxi Cardiovascular Institute, Nanning, Guangxi, China; ^2^Hypertension Division, The First Affiliated Hospital of Guangxi Medical University, Nanning, Guangxi 530021, China

## Abstract

Several studies have indicated that long noncoding RNAs (lncRNAs)-HOX transcript antisense RNA (HOTAIR) is involved in some cardiovascular diseases by regulating gene expression as a competitive endogenous RNA (ceRNA). GJA1 encoding Cx43 is one potential target gene of microRNA-613 (miR-613). Meanwhile, there is a potential target regulatory relationship between HOTAIR and miR-613. The present study is aimed at investigating whether HOTAIR functions as a ceRNA to regulate the Cx43 expression in atrial fibrillation (AF) by sponging miR-613. The expressions of HOTAIR, miR-613, and Cx43 were detected in the right atrial appendages of 45 patients with heart valve disease, including 23 patients with chronic AF. The HOTAIR overexpressed and underexpressed HL-1 cell model were constructed to confirm the effect of HOTAIR on Cx43. Then, the Cx43 expression was detected to testify the interplay between HOTAIR and miR-613 after cotransfecting HOTAIR and miR-613. Furthermore, luciferase assays were performed to verify that HOTAIR could regulate Cx43 remolding as a ceRNA by sponging miR-613. The expression of HOTAIR and Cx43 was significantly downregulated in chronic AF group. HOTAIR regulated positively the Cx43 expression in HL-1 cells. The upregulated effect of HOTAIR on the Cx43 expression could be remarkably attenuated by miR-613. Moreover, the inhibitory effect of miR-613 on the Cx43 expression could be obviously mitigated by HOTAIR. At last, luciferase assays confirmed HOTAIR functioned as a ceRNA in the Cx43 expression by sponging miR-613. Our study suggests that HOTAIR, functioning as a ceRNA by sponging miR-613, is an important contributor to Cx43 remolding in AF.

## 1. Introduction

Atrial fibrillation (AF) is the most common clinical tachyarrhythmia, which is associated with increased risks of stroke, dementia, heart failure, and myocardial infarction [1]. AF affects approximately 33 million people in worldwide [1]. However, the pathogenesis of AF is still not fully understood, which limits the effect of medical intervention on AF.

The focal ectopic firing and reentry are thought to be the two major determinants of AF initiation and perpetuation [2]. The slow conduction velocity and shortened refractoriness promote the reentry [3]. The gap junction, which connects the cytoplasm of adjacent cells, is a key regulator of conduction in the heart [4]. Gap junctions are clustered channels, consisting of two hemichannels, each of which is formed by six connexins (Cxs) [5]. Cx43 is one of the most important Cxs in mammalian atria [5]. A recent study suggested absence of Cx43 could slow down conduction velocity in cardiomyocyte [3, 4]. Tuomi et al. [4] found that the transgenic mice of Cx43 g60s mutant were more likely to induce atrial arrhythmia. Igarashi et al. [6] have demonstrated that gene therapy with adenovirus expressing Cx43 improves atrial conduction and prevents AF in a swine model with atrial pacing. These studies indicated interventions targeting Cx43 may be promising for treatment of AF. However, the underlying regulatory mechanisms of Cx43 in AF remain to be fully elucidated.

MicroRNAs (miRNAs) are a class of small, noncoding RNAs with the length of about 18-25 nucleotides, which negatively regulates the expression of target genes by binding to a complementary sequence in the 3′-untranslated regions (3′-UTRs) of the target mRNAs to promote them degradation or repress them translation [7]. Many studies have shown that dysregulation of miRNAs was associated with cardiovascular diseases, such as arrhythmia [8], myocardial infarction [9], and heart failure [10]. MiR-613 is known to be involved in gastric cancer, colon cancer, and Alzheimer's disease [11–13], but the role of it in Cx43 remolding in AF is still unknown.

Long noncoding RNAs (lncRNAs), a class of RNAs with a length more than 200 nucleotides, are key players in transcriptional regulation and epigenetic gene regulation in cardiovascular diseases [14]. It is the one of the important ways to regulate gene expression that lncRNAs function as a competitive endogenous RNA (ceRNA) by sponging miRNAs [15, 16]. The ceRNA mechanism was proposed by Salmena et al. [17] in 2011. In this hypothesis, messenger RNAs, lncRNAs, and circRNAs can “talk” to each other by binding to shared miRNAs using miRNA response elements [18]. This kind of targeted binding mode can suppress miRNAs like a “sponge” and ultimately inhibit the regulation of miRNAs on downstream target genes. Recently, increasing studies indicated that lncRNAs were associated with AF [19]. Moreover, some lncRNAs, as the ceRNAs by sponging miRNAs, contributed to AF [20]. lncRNA-HOX transcript antisense RNA (HOTAIR) was involved in many cardiac diseases, such as myocardial infarction [21] and congenital heart disease [22]. However, the role of HOTAIR in AF is still unclear. Several studies have indicated HOTAIR regulated gene expression as a ceRNA in some diseases [23]. In addition, the new evidence supported that HOTAIR could sponge miR-613 and upregulate the expression of its downstream target genes [24]. Therefore, the present study is aimed at testifying the hypothesis that HOTAIR could function as a ceRNA to regulate the Cx43 remodeling in AF by sponging miRNA-613.

## 2. Materials and Methods

### 2.1. Human Atrial Samples

A total of 45 patients with valvar heart disease undergoing cardiac surgery were divided into a chronic AF group (23 cases with long-standing persistent AF) and a sinus rhythm (SR) group (22 cases). The characteristics of 45 patients who were included in the current research are summarized in Table 1. The diagnosis of chronic AF was reached by evaluating medical records and 12-lead electrocardiogram findings by two cardiologists. The surgery indication for enrolled patients was in accordance with 2017 ESC/EACTS Guidelines for the management of valvular heart disease [25] and was confirmed by two experienced cardiothoracic surgeons. Those who had hypertension, diabetes mellitus, coronary artery disease, infective endocarditis, tumor, hematological disease, active rheumatism, pulmonary disease, hyperthyroidism, or autoimmune disease were excluded from this study. The protocol was conducted under the Helsinki Declaration and was approved by the Human Ethics Committee of the First Affiliated Hospital of Guangxi Medical University. All patients enrolled in this study gave written informed consent. Right atrial appendage (RAA) tissues were collected at the beginning of the surgical interventions under extracorporeal circulation. Then, the tissues were snap frozen in liquid nitrogen for further biochemical studies.

### 2.2. Predicting Target of miR-613

The target mRNAs of miR-613 were predicted by using the following four softwares: TargetScan (http://www.targetscan.org/), miRDB (http://mirdb.org/), DIANA (http://www.microrna.gr/microT), and miRanda (http://www.microrna.org/microrna/home.do). Those consistently identified by the four databases were regarded as potential target mRNAs of miR-613, and the Venn map was drawn based on the results by online Draw Venn Diagram tool (http://bioinformatics.psb.ugent.be/webtools/Venn/). After prediction, the sequences of potential target genes with high score were analyzed by the National Biotechnology Information Center BLAST program.

### 2.3. Immunohistochemical Staining

The frozen RAA tissues were embedded in optimum cutting temperature compound (OCT) and sectioned at -25°C in a cryostat. Sections (3 *μ*m in thickness) were fixed with 4% polyformaldehyde for 15 minutes at room temperature. Then, the sections were incubated with anti-Cx43 antibody (Cell Signaling Technology, Danvers, MA, USA) diluted 1 : 150 overnight at 4°C. After incubation with the primary antibodies, we washed the sections 3 times at room temperature for 5 minutes each time. Subsequently, the sections were incubated with a secondary antibody (Cell Signaling Technology, Danvers, MA, USA) diluted 1 : 500 for 2 hours at room temperature. Finally, the immunohistochemical staining images were captured by optical microscope (Olympus, Tokyo, Japan) and further analyzed by ImagePro Plus 6.0 (Media Cybernetics, Bethesda, MA, USA).

### 2.4. Cell Culture and Transfection

HL-1, mouse atrial cell line, was purchased from Merck & Co. Ltd (Kenilworth, New Jersey, USA). After HL-1 cells were resuscitated, the cells were cultured in DMEM (Gibco, Gaithersburg, USA) supplemented with 10% fetal bovine serum (FBS), 100 U/ml penicillin, and 100 mg/ml streptomycin. After culture for 24 hours, all the cells were observed under a microscope. If the cells were cultured to about 70%-80% view of 6-cell-culture disc, they were used for transfection of lentivirus or miRNA with Lipofectamine 3000 Reagent (Invitrogen, Carlsbad, CA, USA) according to the manufacturer's instruction. The lentivirus containing HOTAIR, lentivirus containing HOTAIR siRNA, vehicle, miR-613 mimics, and miR-613 mimics negative control (NC) were purchased from GenePharma Co. Ltd (Shanghai, China). All sequences related to transfection are listed in Table 2. The transfected cells were cultured in serum-free medium for 24 hours and then harvested for further experiments. Firstly, to verify the effect of HOTAIR on the Cx43 expression, Cx43 was measured, respectively, in HL-1 cells transfected with vehicle, lentivirus containing HOTAIR, lentivirus containing HOTAIR NC, lentivirus containing HOTAIR siRNA, or lentivirus containing HOTAIR siRNA NC. Then, to testify the interplay between HOTAIR and miR-613, we designed functional rescue and inhibition tests of Cx43. In detail, Cx43 was detected, respectively, in HL-1 cells cotransfected with miR-613 mimics, lentivirus containing HOTAIR, and their associated NC.

### 2.5. Real-Time Quantification PCR (RT-qPCR) Analysis

Total RNA was extracted from tissues and cells with TRIzol reagent (Invitrogen, Carlsbad, CA, USA). The miRNAs were extracted from tissues with RNAiso for Small RNA reagent (Takara, Tokyo, Japan). Total RNAs were reverse-transcribed into complementary cDNA using a PrimerScript™ RT reagent kit with a gDNA eraser (Takara, Tokyo, Japan) according to the manufacturer's protocols. The PCR procedure was as follows: one cycle at 95°C for 30 seconds, followed by 40 cycles of 95°C for 5 sec and 60°C for 31 seconds. Melting curve analysis was performed at 65~ 95°C. The miRNAs were polyadenylated and subsequently converted into cDNAs using a Mir-X™ miRNA First-Strand Synthesis kit (Clontech Laboratories, Mountain View, CA, USA) according to the manufacturer's protocols. The PCR procedure was identical to that mentioned above. All the data obtained were calculated by the 2^-*ΔΔ*Ct^ method. We used GAPDH as an internal reference for HOTAIR and Cx43. U6 was used as the internal standard for normalizing gene expression of miR-613. All primer sequences for RT-qPCR are listed in Table 2.

### 2.6. Western Blot Analysis

Total proteins were extracted from tissues and cells in RIPA buffer (Beyotime Institute of Biotechnology, Shanghai, China) with PMSF (Sigma-Aldrich, St. Louis, MO, USA). The total protein concentration was measured by BCA protein assay kit (Beyotime Institute of Biotechnology, Shanghai, China). The proteins were boiled with 5x SDS-PAGE loading buffer (Solarbio, Beijing, China) and separated by 10% sodium dodecyl sulfated-polyacrylamide gel electrophoresis and then transferred to 0.22 *μ*m of PVDF membrane (Millipore, Billerica, MA, USA) using the Mini Trans-Blot electrophoretic transfer cell system (Bio-Rad, Hercules, CA, USA). The membranes were blocked for 1 h at room temperature with 5% nonfat milk in TBST (20 mM Tris-HCl, 0.5 M NaCl, 0.1% Tween 20) and incubated with anti-Cx43 antibody (Abcam, Cambridge, UK) diluted 1 : 1,000 and anti-GAPDH antibody (Abcam, Cambridge, UK) diluted 1 : 10000 overnight at 4°C. Subsequently, the membranes were incubated with IRDye® 800CW goat anti-rabbit IgG or goat anti-mouse IgG (LI-COR Biotechnology, Lincoln, NE, USA) diluted at 1 : 10,000 for 1 hour at room temperature. The signals were detected and quantified with the Odyssey system (LI-COR Biotechnology, Lincoln, NE, USA). Protein band intensities were expressed relative to GAPDH.

### 2.7. Luciferase Activity Assays

To testify whether GJA1 is a direct target gene of miR-613, the 3′UTR sequences of GJA1 containing a binding site of miR-613 and its mutants were cloned into the luciferase reporter plasmid pmirGLO to construct a wild-type (GJA1-WT) and mutant (GJA1-Mut) recombinant luciferase reporter plasmids, respectively. Subsequently, HEK293T cells were cotransfected with GJA1-WT recombinant luciferase reporter plasmids or GJA1-Mut recombinant luciferase reporter plasmids and miR-613 mimics or miR-613 mimics NC. To verify whether HOTAIR competes with GJA1 for miR-613 binding, the cells were cotransfected with GJA1-WT recombinant luciferase reporter plasmids and miR-613 mimics, miR-613 mimics NC, miR-613 mimics+lentivirus containing HOTAIR, or miR-613 mimics+lentivirus containing HOTAIR NC. The luciferase activity was measured with dual-luciferase Reporter Assay System (Promega, Madison, WI, USA) and analysis at 24 hours after transfection.

### 2.8. Statistical Analysis

In our article, for continuous data, the normal distribution test was carried out first. If the data did not belong to normal distribution, we would conduct logarithmic processing on the data before data analysis. The continuous data are presented as mean ± standarddeviation (SD). Meanwhile, SD is showed as error bars in the charts of our article. The continuous variables between 2 groups were analyzed by the unpaired Student *t*-test or Mann–Whitney *U* test and among multiple groups using one-way ANOVA followed by Holm-Sidak's test. The discrete data are shown as percentages. The discrete variables between 2 groups were analyzed by chi-squared test. A *P* value < 0.05 was considered statistically significant. All statistical analyses were performed using SPSS 20.0 (SPSS Inc., Chicago, Ill., USA).

## 3. Results

### 3.1. HOTAIR Is Involved in Cx43 Remolding in AF

**Figure 1:**
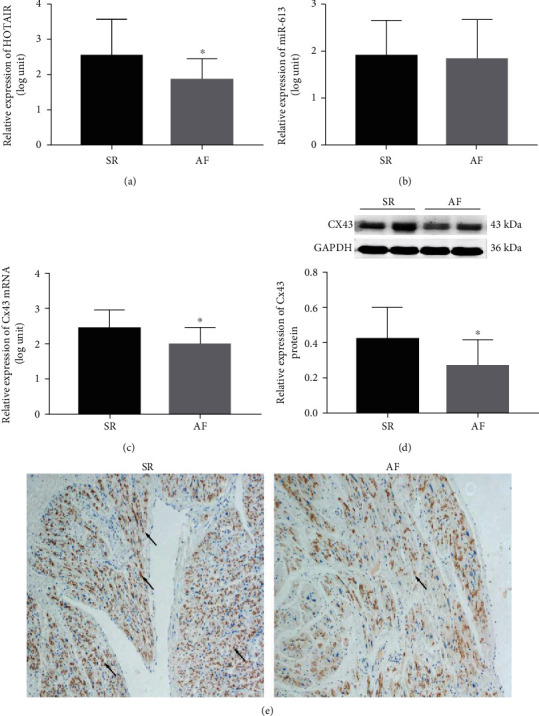
Downregulation of Cx43 and HOTAIR in RAA of patients with chronic AF. (a) The expression of HOTAIR in RAA of patients with SR or chronic AF. (b) The expression of miR-613 in RAA of patients with SR or chronic AF. (c) The mRNA expression of Cx43 in RAA of patients with SR or chronic AF. (d) Western blot analysis and quantification of Cx43 between the two groups. GAPDH was used as an internal control. (e) Representative images of immunohistochemistry staining of Cx43 (×100) between the two groups. Brown staining represents Cx43. The Cx43 was marked with single arrow. ^∗^*P* < 0.05, against the SR group. SR: sinus rhythm; AF: atrial fibrillation; RAA: right atrial appendage; miR: microRNA; HOTAIR: HOX transcript antisense RNA; Cx: connexin; GAPDH: glyceraldehyde-3-phosphate dehydrogenase. The error bars mean standard deviation.

The expression of HOTAIR in chronic AF group was significantly downregulated compared to the SR group by RT-qPCR (*P* < 0.05; Figure 1(a)). However, compared with patients in the SR group, there was no significant difference in the miR-613 expression in RAA tissues of patients with chronic AF (*P* > 0.05; Figure 1(b)). The Cx43 mRNA level and the Cx43 protein level in the chronic AF group were significantly downregulated (*P* < 0.05; Figures 1(c) and 1(d)). Consistently, the reduced Cx43 expression was detected in the chronic AF group by IHC (*P* < 0.05; Figure 1(e)). These results indicated that HOTAIR may be positively associated with the Cx43 expression.

### 3.2. Prediction of miR-613 Target RNAs

**Figure 2:**
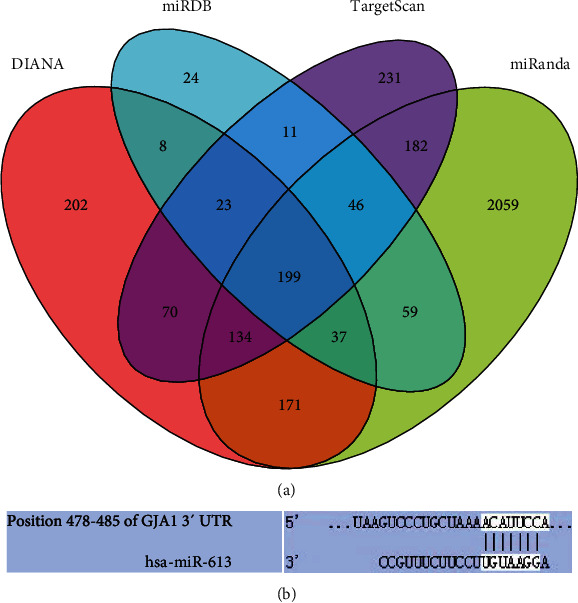
GJA1 is a potential downstream target gene of miR-613: (a) the Venn map of the potential target genes of miR-613; (b) predicted binding site of miR-613 to GJA1 mRNA in Targetscan.

After preliminary bioinformatics predictions, we found 199 target genes for miR-613 totally (Figure 2(a)), and there was one potential binding site with miR-613 in 3′-UTR of GJA1 mRNA, which indicated that GJA1 may be a putative target gene of miR-613 (Figure 2(b)). Interestingly, some evidences have revealed that HOTAIR is a direct target RNA. Therefore, we speculated that HOTAIR might compete with GJA1 for miR-613 binding to promote the Cx43 expression.

### 3.3. HOTAIR Positively Regulates Cx43 by Attenuating Suppressive Effect of miR-613 on Cx43 Expression

**Figure 3:**
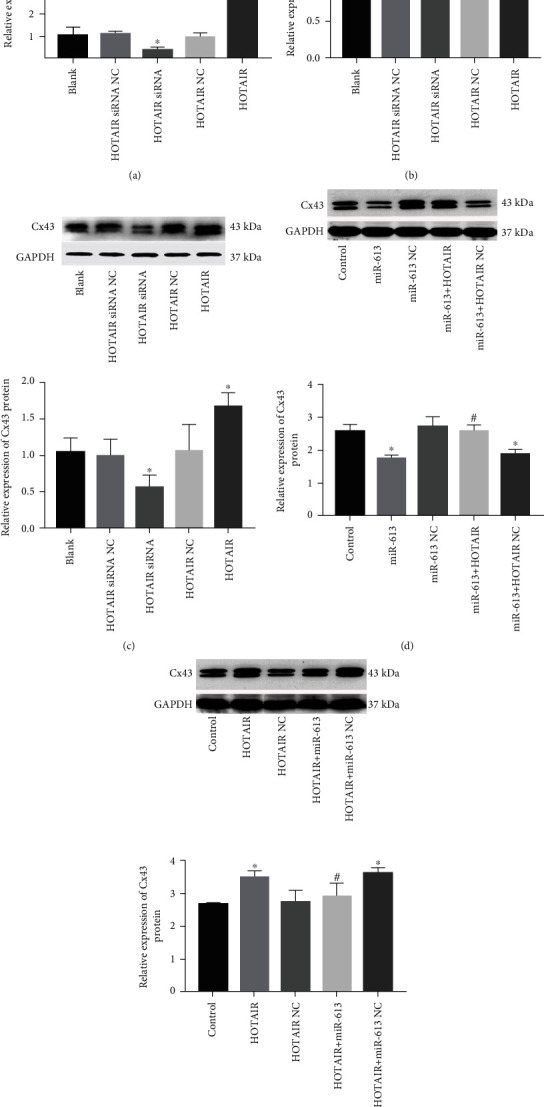
HOTAIR positively regulates Cx43 by attenuating suppressive effect of miR-613 on the Cx43 expression. (a) The efficacy evaluation of the expression vector of HOTAIR and HOAIR siRNA after transfection; ^∗^*P* < 0.05, against the blank group, HOTAIR siRNA NC group, or HOTAIR NC group. (b) HOTAIR has no direct effect on the miR-613 expression in HL-1 cell. (c) HOTAIR positively regulates the Cx43 expression in HL-1 cell. GAPDH was used as an internal control; ^∗^*P* < 0.05, against the blank group, HOTAIR siRNA NC group, or HOTAIR NC group. (d) HOTAIR counteracts the suppressive effect of miR-613 on the Cx43 expression. GAPDH was used as an internal control; ^∗^*P* < 0.05, against the control group; ^#^*P* < 0.05, against the miR-613 group or miR-613 + HOTAIR NC group. (e) miR-613 counteracts the upregulated effect of HOTAIR on the Cx43 expression. GAPDH was used as an internal control; ^∗^*P* < 0.05, against the control group; ^#^*P* < 005, against the HOTAIR group or HOTAIR+miR-613 NC group. miR: microRNA; HOTAIR: HOX transcript antisense RNA; Cx: connexin; GAPDH: glyceraldehyde-3-phosphate dehydrogenase. The error bars mean standard deviation.

To testify whether HOTAIR was involved in the regulation of Cx43 in cardiomyocytes, a HOTAIR knockdown or overexpressed HL-1 cell model was made. After transfection, RT-qPCR results revealed that HOTAIR siRNA suppressed the expression of HOTAIR (*P* < 0.05; Figure 3(a)). By contrast, lentivirus containing HOTAIR significantly increased the expression of HOTAIR (*P* < 0.05; Figure 3(a)). There was no significant difference in expression of miR-613 among each group (*P* > 0.05; Figure 3(b)), suggesting HOTAIR was not directly involved in the regulation of the miR-613 expression. Western blot results showed that the Cx43 protein level significantly increased in the HOTAIR group, while it markedly decreased in the HOTAIR siRNA group (*P* < 0.05; Figure 3(c)). There was no significant difference in Cx43 protein level among control, HOTAIR NC group, and HOTAIR siRNA NC group. These evidences indicated that HOTAIR positively regulated the Cx43 expression in HL-1 cells.

To further investigate how HOTAIR positively regulates the Cx43 expression in cardiomyocytes, the HL-1 cells were transfected with miR-613 mimics, miR-613 mimics NC, miR-613 mimics+lentivirus containing HOTAIR, or miR-613 mimics+lentivirus containing HOTAIR NC, respectively. The Cx43 protein level was measured in each group. The results showed that the Cx43 protein level in the miR-613 group was significantly decreased compared to those in the control and miR-613 NC group, but the Cx43 protein level in the miR-613 + HOTAIR group was markedly elevated compared to those in the miR-613 group (*P* < 0.05; Figure 3(d)). Then, the HL-1 cells were transfected with lentivirus containing HOTAIR, lentivirus containing HOTAIR NC, lentivirus containing HOTAIR+miR-613 mimics, or lentivirus containing HOTAIR+miR-613 mimics NC, respectively. The Cx43 protein level was detected in each group. The results showed that the Cx43 protein level in the HOTAIR group was significantly increased compared to those in the control and HOTAIR NC group, but the Cx43 protein level in the HOTAIR+miR-613 group was markedly decreased compared to those in the HOTAIR group or the HOTAIR+miR-613 NC group (*P* < 0.05; Figure 3(e)).

All of the above reveal that there is a competitive relationship in regulation of Cx43 between HOTAIR and miR-613, and HOTAIR positively regulates Cx43 by attenuating suppressive effect of miR-613 on the Cx43 expression.

### 3.4. HOTAIR May Regulate the Expression of Cx43 by Sponging miR-613 as a ceRNA

**Figure 4:**
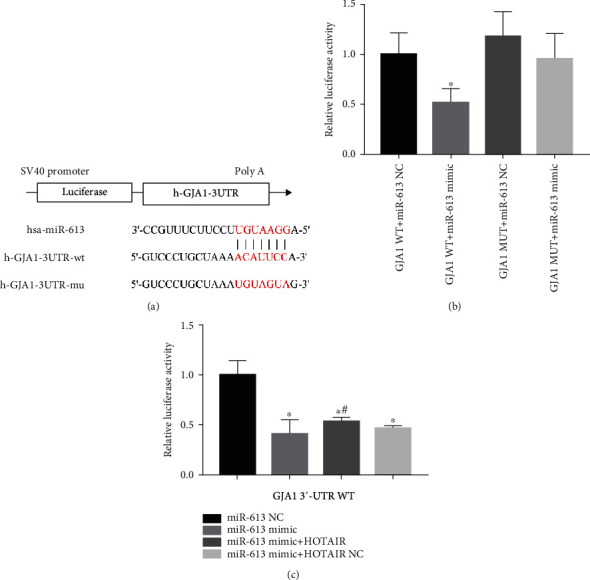
Two-step luciferase assays confirmed HOTAIR may directly competing with GJA1 for miR-613 binding and positively regulate the Cx43 expression by sponging miR-613 as a ceRNA. (a) The binding site of miR-613 and GJA1 3′UTR. (b) GJA1 is a direct downstream target of miR-613; ^∗^*P* < 0.05, against the GJA1 WT + miR-613 NC group. (c) HOTAIR may directly competing with GJA1 for miR-613 binding; ^∗^*P* < 0.05, against the miR-613 NC group; ^#^*P* < 0.05, against the miR-613 mimic group. miR: microRNA; HOTAIR: HOX transcript antisense RNA; UTR: untranslated regions; ceRNA: competitive endogenous RNA. The error bars mean standard deviation.

To testify whether HOTAIR might directly compete with GJA1 for miR-613 binding to promote the Cx43 expression, two-step luciferase assays were performed. In first-step luciferase assays, we testified whether GJA1 was a direct target gene of miR-613. The binding site of miR-613 and GJA1 3′UTR is shown in Figure 4(a). Luciferase assay results showed that miR-613 was able to suppress the luciferase activity of the GJA1-WT recombinant luciferase reporter plasmids. However, GJA1-Mut recombinant luciferase reporter plasmid was not able to be inhibited by miR-613 (*P* < 0.05; Figure 4(b)). These results demonstrate that GJA1 is a direct downstream target of miR-613, and HOTAIR has a structural basis to compete with GJA1 for miR-613 binding. In second-step luciferase assays, we testified the competitive relationship between HOTAIR and GJA1 for miR-613 binding. GJA1-WT recombinant luciferase reporter plasmids were cotransfected with miR-613 mimics NC, miR-613 mimics, miR-613 mimics+lentivirus containing HOTAIR, or miR-613 mimics+lentivirus containing HOTAIR NC in HEK293T cells, respectively. The results showed that miR-613 significantly suppressed luciferase activity of GJA1-WT recombinant luciferase reporter plasmids, but the inhibitory effect of miR-613 on luciferase activity of GJA1-WT recombinant luciferase reporter plasmids was counteracted by HOTAIR (*P* < 0.05; Figure 4(c)). These findings suggest that HOTAIR may directly competing with GJA1 for miR-613 binding and positively regulate the Cx43 expression by sponging miR-613 as a ceRNA.

## 4. Discussion

At present, a number of achievements have been made in cardiological treatment, but the risk of AF remains to affect people's lives and increases with age. Of note, some recent studies on lncRNAs reveal that dysregulation of lncRNAs is relative to the pathogenesis of AF [26–29]. lncRNAs is considered as a promising target for AF intervention. To our best knowledge, this is the first study to elucidate the specific effect of HOTAIR on regulation of the Cx43 expression in atrial electrical remodeling of AF via acting as ceRNA to sponge miR-613.

In our study, the baseline characteristics between patients with SR or AF have no obvious difference, except for the increase of left atrium (LA) diameter in patients with AF. In accordance with our study, echocardiography result of ENGAGE AF-TIMI 48 study also found the enlargement of LA and reduced left atrial emptying fraction in 55% of patients with AF [29]. LA enlargement with a consequent decrease in LA function represents maladaptive structural and functional remodeling that in turn promotes electrical remodeling and a milieu conducive for incident AF [30, 31].

lncRNAs have been found to have multiple biological functions related to transcriptional, posttranscriptional, translational, and epigenetic gene regulation [32]. HOTAIR locates in chromosome 12 between HOXC11 and HOXC12. HOTAIR was found to be associated with numerous cardiovascular diseases. Gao et al. [21] demonstrated that the expression of HOTAIR decreased in the serum of patients with acute myocardial infraction. Greco et al. [33] reported that HOTAIR was downregulated obviously in patients with ischemic heart failure. Lai et al. [34] found that downregulation of HOTAIR was associated with ventricular hypertrophy in transverse aortic constriction rat model. Zhang et al. [35] reported overexpression of HOTAIR could decrease myocardial infraction size and the level of myocardial necrosis marker in the coronary artery ligation model of rats. Taken together, the downregulation of HOTAIR is involved in the process of different cardiac diseases, suggesting HOTAIR may act as a protective role in cardiovascular diseases. Similar to the above studies, we found that a decreased expression of HOTAIR in RAA of patients with chronic AF, suggesting that the downregulated HOTAIR may promote the occurrence of AF.

On the other hand, the protein level of Cx43, which was decreased in patients with chronic AF, was positively correlated with expression level of HOTAIR, indicating HOTAIR may be involved in the Cx43 remodeling in AF. The downregulation of Cx43 is linked to atrial electrical remodeling, which has been confirmed in both animal models and human tissue studies. Igarashi et al. [6] observed that the expression of Cx43 in a pig model of AF was downregulated. Absence of Cx43 could slow down conduction velocity among cardial myocytes, which leaded to the nonuniformity of electrical conduction and the decreased of electrical coupling. In our previous study, we found that heteromorphic Cx43 could reduce cell dispersion and monomorphic conductivity in heart [36]. All this change induced by abnormal Cx43 forms the pathological basis of electrical remolding and increases the inductivity of AF. The quantity and spatial distribution of atrial Cx43 could be restored via gene intervention with adenovirus expressing Cx43, which preserves atrial conduction and prevents AF in a rapid atrium pacing swine model [6].

In order to detect the potential relationship between HOTAIR and Cx43, we used HOTAIR or HOTAIR siRNA to transfect HL-1 cells. Western blot results confirmed that HOTAIR could positively regulate the expression of Cx43. But how HOTAIR regulates the expression of Cx43 in detail is still unknown. Interestingly, GJA1 encoding Cx43 is predicted as a potential target of miR-613. In our study, the Cx43 protein level was significantly downregulated by miR-613 mimics, and the luciferase activity of GJA1-WT recombinant luciferase reporter plasmids was obviously suppressed by miR-613 mimics. These results suggest that GJA1 encoding Cx43 was a direct target of miR-613 and miR-613 negatively regulate the Cx43 expression at the level of posttranscription.

Increased evidence indicated that lncRNAs can interact with miRNAs to regulate the expression of their downstream target genes at the level of posttranscription, as “molecular sponges” weakening negatively regulatory effect of miRNAs on their target genes [37, 38]. Recently, HOTAIR was proposed to function as a ceRNA by sponging miRNAs to regulate target gene expression of miRNAs. For example, HOTAIR could competitively bind miR-331-3p to regulate the HER2 expression in gastric cancer [39]. In the present study, the inhibitory effect of miR-613 on the Cx43 expression was partially attenuated by HOTAIR. Meanwhile, miR-613 mimics counteracted the positive regulation of HOTAIR on Cx43. These results preliminarily indicate that HOTAIR may regulate Cx43 by competing with GJA1 for miR-613 binding. Because there have been studies that demonstrate HOTAIR directly targets miR-613 [40, 41], we did not repeat luciferase assay to verify whether HOTAIR is a target of miR-613. We focused on competitive relationship for miR-613 binding between GJA1 encoding Cx43 and HOTAIR. Therefore, another luciferase assay was performed. The luciferase activity was observed in HEK 293T cells cotransfected with GJA1-WT recombinant luciferase reporter plasmids and miR-613 mimics, or GJA1-WT recombinant luciferase reporter plasmids and miR-613 mimics+HOTAIR. We found that miR-613 significantly suppressed luciferase activity of GJA1-WT recombinant luciferase reporter plasmids, but the inhibitory effect of miR-613 on luciferase activity of GJA1-WT recombinant luciferase reporter plasmids was counteracted by HOTAIR. These findings further verify that HOTAIR regulates the Cx43 expression by sponging miR-613 directly. Of note, there was no significant difference in expression of miR-613 among each group. This suggests that HOTAIR was not directly involved in the regulation of miR-613. This evidence also supports the insight that HOTAIR regulates Cx43 by sponging miR-613.

Our study provided an important insight into how HOTAIR specifically interacts with miR-613 in Cx43 remodeling of AF. However, there are some limitations to our study. Because of the small size sample that only included valvular heart disease patients with SR or chronic AF, the regulatory effect of HOTAIR on Cx43 could not be evaluated in different types of heart disease. Additionally, animal models were in demand to verify the results drawn from this investigation in vivo.

In conclusion, the present study found the decreased expression of HOTAIR in patient with AF and HOTAIR positively regulated Cx43 remolding in AF via acting as a ceRNA by sponging miR-613. The HOTAIR/miR-613/Cx43 axis may be a novel promising intervention target for electrical remodeling of AF. However, further investigations are still required to verify this consequence in vivo.

## Figures and Tables

**Table 1:** The baseline characteristics of included patients.

	SR (22)	AF (23)	*P* value
Gender (male/female)	11/11	7/16	0.181
Age (year)	52.14 ± 12.30	51.17 ± 8.52	0.761
Average duration of AF (year)	—	2.66 ± 1.20	—
Echocardiography			
LAd (mm)	44.45 ± 7.58	53.43 ± 11.21	0.003^∗^
LVEDd (mm)	57.23 ± 10.31	51.57 ± 9.36	0.060
LVESd (mm)	37.41 ± 7.97	34.39 ± 8.29	0.221
LVEF (%)	62.55 ± 10.18	60.70 ± 7.60	0.492
Valvular surgery type			
AVR	4	1	0.187
MVR	12	16	0.299
Combined valve replacement	6	6	0.928
Therapy			
*β*-Blockers (*n*)	8	7	0.458
Digoxin (*n*)	3	11	0.013^∗^
Amiodarone (*n*)	—	10	—

SR: sinus rhythm; AF: atrial fibrillation; LA: left atrial diameter; LVED: left ventricular end-diastolic diameter; LVES: left ventricular end-systolic diameter; LVEF: left ventricular ejection fraction; AVR: aortic valve replacement; MVR: mitral valve replacement. ^∗^*P* < 0.05, against the SR group.

**Table 2:** The sequence list.

Subject	Sequence	
Primer sequence for RT-qPCR		
HOTAIR	Forward	5′-GGTCCCTAATATCCCGGAGGTG-3′
	Reverse	5′-GCAGGCTTCTAAATCCGTTCCA-3′
Cx43	Forward	5′-CTCTCGCCTATGTCTCCTCCT-3′
	Reverse	5′-GTTTTGCTCACTTGCTTGCTT-3′
GADPH	Forward	5′-GCACCGTCAAGGCTGAGAAC-3′
	Reverse	5′-TGGTGAAGACGCCAGTGGA-3′
U6	Forward	5′-CTCGCTTCGGCAGCACA-3′
	Reverse	5′-AACGCTTCACGAATTTGCGT-3′
miR-613	Forward	5′-AGGAATGTTCCTTCT-3′
	Reverse	5′-GTGCAGGGTCCGAGGT-3′
Target gene sequence for transfection		
miR-613 mimic	Sense	5′-AGGAAUGUUCCUUCUUUGCC-3′
	Antisense	5′-CAAAGAAGGAACAUUCCUUU-3′
miR-613 mimic NC	Sense	5′-UUCUCCGAACGUGUCACGUTT-3′
	Antisense	5′-ACGUGACACGUUCGGAGAATT-3′
HOTAIR siRNA	Sense	5′-GCTGACATACATGGCTATTTCT-3′
	Antisense	5′-AGAAATAGCCATGTATGTCAGC-3′

## Data Availability

All the data used to support the findings of this study were supplied by Zhiyuan Jiang under license. Requests for access to these data should be made to Weiran Dai (e-mail: daiweiran2008@163.com).
